# Comprehensive Analysis of Soluble Mediator Profiles in Congenital CMV Infection Using an MCMV Model

**DOI:** 10.3390/v16020208

**Published:** 2024-01-30

**Authors:** Dubravka Karner, Daria Kvestak, Berislav Lisnic, Maja Cokaric Brdovcak, Vanda Juranic Lisnic, Paola Kucan Brlic, Milena Hasan, Tihana Lenac Rovis

**Affiliations:** 1 Center for Proteomics, Faculty of Medicine, University of Rijeka, 51000 Rijeka, Croatia; dubravka.karner@uniri.hr (D.K.); daria.kvestak@medri.uniri.hr (D.K.); berislav.lisnic@uniri.hr (B.L.); maja.cokaric@medri.uniri.hr (M.C.B.); vanda.juranic@medri.uniri.hr (V.J.L.); paola.kucan@medri.uniri.hr (P.K.B.); 2 Cytometry and Biomarkers Unit of Technology and Service (CB TechS), Institut Pasteur, Université Paris Cité, 75015 Paris, France; milena.hasan@pasteur.fr

**Keywords:** congenital human cytomegalovirus infection, HCMV, MCMV, cytokine, chemokine

## Abstract

Congenital human cytomegalovirus (HCMV) infection may cause life-threatening disease and permanent damage to the central nervous system. The mouse model of CMV infection is most commonly used to study mechanisms of infection and pathogenesis. While essential to limit mouse CMV (MCMV) replication, the inflammatory responses, particularly IFNγ and TNFα, cause neurodevelopmental abnormalities. Other soluble mediators of the immune response in most tissues remain largely unexplored. To address this gap, we quantified 48 soluble mediators of the immune response, including 32 cytokines, 10 chemokines, 3 growth factors/regulators, and 3 soluble receptors in the spleen, liver, lungs, and brain at 9 and 14 days postinfection (dpi). Our analysis found 25 induced molecules in the brain at 9 dpi, with an additional 8 showing statistically elevated responses at 14 dpi. Specifically, all analyzed CCL group cytokines (CCL2, CCL3, CCL4, CCL5, CCL7, and CCL11) were upregulated at 14 dpi in the brain. Furthermore, data revealed differentially regulated analytes across tissues, such as CCL11, CXCL5, and IL-10 in the brain, IL-33/IL-33R in the liver, and VEGF-a and IL-5 in the lungs. Overall, this study provides an overview of the immune dynamics of soluble mediators in congenital CMV.

## 1. Introduction

While the immunocompetent population can manage primary human CMV (HCMV) infection with relative ease, the immunocompromised demographic faces a spectrum of severe complications, including encephalitis, pneumonitis, and hepatitis [1,2]. Of particular concern is congenital HCMV infection, which encompasses a range of impairments such as microcephaly, hearing loss, cerebral palsy, mental retardation, seizures, ocular abnormalities, and cognitive impairment [1,3,4,5]. Vulnerability in both contexts arises from the absence of robust defense mechanisms present in a fully developed immune system. Addressing congenital HCMV infection presents a challenge since currently available antivirals have serious side effects and there is growing concern regarding escape mutants [6,7]. Development of new and better therapies will rely on the proper understanding of the disease and immune dynamics in congenital cases, paving the way for targeted therapeutic interventions tailored to their specific challenges.

A significant challenge in unraveling the pathogenesis of HCMV infection lies in its strict species specificity, confining investigations to animal models [8]. The mouse CMV (MCMV) is a pivotal model in HCMV research, offering valuable insights into various facets of HCMV disease [8,9,10,11]. One drawback of the mouse model is that, unlike HCMV, MCMV does not cross the placenta. Nevertheless, this limitation has been successfully circumvented by several models [12] that faithfully recapitulate pathologies and clinical outcomes observed in humans. In this study, we use a congenital infection model wherein newborn mice are intraperitoneally (i.p.) infected with MCMV on postnatal day 1 [13]. Notably, this model faithfully recapitulates key aspects of central nervous system (CNS) infections observed in second/third trimester human fetuses, mirroring the route of viral neuroinvasion and corresponding neuropathological findings [13,14,15]. Following i.p. inoculation, MCMV undergoes initial replication in peripheral organs before disseminating to the CNS, where the infectious virus is first detected on postnatal day 6/7. Virus titers peak in the CNS between postnatal days 10 and 14, with the infectious virus becoming undetectable by day 21 [14,15,16]. Adult mice, like humans, do not manifest brain infections. Considering the devastating impact of newborn brain infection, the research in the congenital MCMV model is predominantly concentrated on brain pathogenesis, with other CMV-infected tissues remaining largely unexplored.

The intricacies of CMV infection unfold through a dynamic interplay of immune responses, marked by the robust induction of proinflammatory cytokines [13,14,15,16,17,18,19]. Notably, the study by Ruzek et al. highlights the rapid surge of cytokines such as IL-12, TNF, IFN-γ, and IL-1α in adult mice following i.p. MCMV infection [18]. This inflammatory response mirrors observations in i.p. infected newborn mice, albeit with delayed kinetics [14,17,20]. In newborn mice, the serum concentration of IFN-γ peaked around 8 dpi, with comparable kinetics observed in the brain, including other proinflammatory cytokines (TNF-α and IL-6) [14,20]. Microarray gene expression analysis revealed a peak in IL1β at day 5 p.i. [13]. Concerning chemokines, existing evidence highlights an increased expression of CXCL9, CXCL10, CCL5, and CCL2 in infected newborn brains [13,14,20]. In the CNS, a robust inflammatory response is characterized by the activation of microglia, infiltration of immune cells, and upregulation of proinflammatory cytokines [13,14,17,20]. The significant impact of these immune responses on cerebellar pathology is underscored by interventions in mice, such as glucocorticoid treatment, NK/ILC1 cell depletion, or IFN-γ/TNFα blockade, which all ameliorate neuroinflammation and mitigate deficits in cerebellar development [14,17,20]. Adding relevance to the immune-mediated findings of MCMV infection, a study by Sellier et al. establishes a link between severe fetal brain damage and elevated NK cells in HCMV-infected fetal brains [21]. The majority of data on cytokines, chemokines, and other relevant inflammation mediators in the congenital infection model rely on gene expression data rather than protein levels and are generally focused on the brain.

In this study, we reveal a profound influence of MCMV infection on the pattern of soluble mediators in newborn mice across different organs and at different time points postinfection. Among the 47 detected molecules involved in the inflammatory response, 16 exhibited alterations during the early phase of acute infection, spanning both the periphery and CNS. Our data present a comprehensive insight into the cytokine and chemokine landscape during congenital CMV infection, contextualizing it within current knowledge. Novel findings include previously unmeasured molecules (e.g., CCL3, CCL4, and CCL7) and organ-specific nuances of cytokine expression (e.g., IL10 in the brain, IL33/IL33R in the liver, or VEGFA in the lungs). These results facilitate navigating through the intricate pattern of immune response and cytokine dynamics involved in the regulation of inflammation in the context of CMV infection across different tissues and time points.

## 2. Materials and Methods

### 2.1. Viruses

All infections were conducted using BAC pSM3fr-derived MCMV (C3X) that had been based on the MCMV Smith strain (ATCC VR-1399). The virus was produced on BALB/c mouse embryonic fibroblasts (MEF), ultracentrifuged (Sorvall), and titrated by standard plaque assay, as previously described [11]. Produced virus stocks were titrated by serial 10-fold dilution in 48-well tissue culture plates containing an MEF monolayer. After overlaying with methylcellulose and 4 days of incubation, we determined PFU per milliliter based on plaque counting.

### 2.2. Mice

C57BL/6J and BALB/c mice were obtained from The Jackson Laboratory. C57BL/6J-Prnp−/− mice PrnpZH3/ZH3 (PrP ko) [22] were a generous gift from A. Aguzzi. Mice were strictly age-matched within experiments and handled in accordance with the guidelines contained in the International Guiding Principles for Biomedical Research Involving Animals. All mice were housed and bred under specific pathogen-free conditions at the Central Animal Facility of the Faculty of Medicine, University of Rijeka. The Ethics Committee at the University of Rijeka and The National Ethics Committee for the Protection of Animals Used for Scientific Purposes (Ministry of Agriculture) approved experiments with laboratory animals (approval number HR-POK-004). For congenital infection, newborn mice were infected intraperitoneally (i.p.) with 400 PFU of MCMV prepared in a volume of 50 µL of pure DMEM on postnatal day 0 (BALB/c) or day 1 (C57BL/6J and PrP ko).

### 2.3. Organ Harvesting for Cytokine Luminex^®^ Performance Assay

At 9 or 14 days postinfection, mice were sacrificed. Brain, spleen, lung, and liver tissue were collected into cryotubes (Greiner Bio-One GmbH, Kremsmünster, Austria), weighed, snap-frozen by dipping the tubes into liquid nitrogen, and stored at −80 °C until further processing.

### 2.4. Cytokine Luminex^®^ Performance Assay 

Cryopreserved organs were lysed using Procartaplex™ buffer (Thermo Scientific, Waltham, MA, USA) and homogenized using steel beads (3 × 30 s at frequency 2.5). An amount of 500 µL of lysis buffer was added per 100 µg of tissue. Tissue proteins were quantified using a ProcartaPlex™ Mouse Immune Monitoring Panel 48-Plex according to manufacturer’s instructions (EPX480-20834-901, Thermo Scientific). Briefly, DropArray 96-well-plates (Curiox Biosystems, Woburn, MA, USA) were blocked with 1% BSA for 30 min. Samples were diluted 1:2. After blocking, 10 µL of premixed beads, followed by 10 µL of samples, controls, or standard were added per well and incubated for 2 h. The plates were incubated on the shaker, with 5 µL of detection antibody for 30 min, 10 µL of streptavidin-PE for 30 min, and 15 µL of reading buffer per well, before being transferred to reading plates. Between incubation steps, plates were washed three times (PBS, 0.1% BSA, 0.05% tween) at a DropArray LT210 MX washing station (Curiox Biosystems). Samples were read by the Bio-Plex200™ instrument (Bio-Rad, Hercules, CA, USA) in 70 µL of reading buffer [23]. 

### 2.5. CBA Kit

Protein levels of IL-10 and CCL2 in brain homogenates were measured by flow cytometry using the CBA Mouse Inflammation Kit (BD Biosciences, San Diego, CA, USA) according to the manufacturer’s protocol. Brains harvested from MCMV-infected and naïve BALB/c mice were lysed in DMEM with cOmplete protease inhibitor (Roche, Basel, Switzerland) with a bead homogenizer. All data were acquired using FACSAriaIIu (BD Biosciences) and analyzed using FlowJo v10 software (Tree Star Inc., Ashland, OR, USA).

### 2.6. Statistical Analysis and Data Interpretation

Data are presented as mean or mean + SEM. Statistical significance was determined by unpaired *t*-test using GraphPad Prism 8 (GraphPad Prism 8 Software, San Diego, CA, USA). A value of *p* > 0.05 was considered not statistically significant (ns): *, *p* < 0.05; **, *p* < 0.01; ***, *p* < 0.001; ****, *p* < 0.0001. When more than half of the samples in the infected group’s results were below the limit of detection and displayed as “OOR<” or out of range below, it was considered nondetectable. With many proinflammatory cytokines not being present in the tissue without the cause, the naïve group of samples was assigned 0 value in the case of “OOR<” so the statistical analysis could still be performed. “OOR>” or out of range above was assigned a value by taking a maximum detectable value of an analyte on the plate and multiplying it by 2 [24,25]. Values that were automatically extrapolated by the software were included in the analysis.

### 2.7. Generation of Clustered Heatmap

Groups of cytokines exhibiting similar or related expression profiles across different time points and organs in MCMV-infected animals were identified by performing K-means clustering using the R computing environment v4.3.2 [26] within the RStudio IDE v2023.9.1, build 494 [27]. Briefly, cytokine concentration data stored in MS Excel files were first loaded into R using the *readxl* package [28]. Following import into R, cytokine concentrations were log(x + 1) transformed, scaled, and used as input for constructing and drawing a clustered heatmap using the *ComplexHeatmap* package [29], setting the *row_km* value to 4, and using the default settings for distance measuring (Euclidean) and clustering method (complete). When required, data wrangling and heatmap customization procedures within R were carried out using the functionalities available in the *tidyverse* v2.0.0 [30], *RColorBrewer* v1.1.3 [31], *scales* v1.3.0 [32], *cowplot* v1.1.1 [33], *circlize* v0.4.15 [34], and *gridtext* v0.1.5 [35] R packages.

## 3. Results and Discussion

### 3.1. In-Depth Analysis of Soluble Immune Mediators during Congenital CMV Infection across Different Organs

In this study, we employed a mouse model of congenital CMV infection to explore 48 inflammatory mediators, encompassing 32 cytokines, 10 chemokines, 3 growth factors/regulators, and 3 soluble receptors. Our objective was to elucidate the dynamic changes in immune and regulatory molecules during CMV infection. We focused on the most commonly studied organs—liver, lungs, spleen, and brain—examining them 9 days after i.p. virus inoculation. The latter two organs underwent scrutiny at both 9 and 14 dpi to track and reflect the later onset and peak of CMV infection in the brain [15]. We successfully detected 47 out of the 48 analyzed molecules at the 9- or 14-dpi time point, with only IL-7 remaining undetected. Our results revealed that 16 analytes were statistically elevated at 9 dpi across all organs. Among them, nine cytokines and six chemokines remained statistically elevated in both the spleen and brain at 14 dpi. The Figshare 1 (https://figshare.com/s/83ba2568c37ebf2eabe8) provides a molecule-by-molecule breakdown for a comprehensive evaluation of each of the 47 identified molecules. In Table 1, we present organ-specific data, depicting statistical significance for measured interleukins, interferons, TNFα, and the CC and CXC chemokines.

Next, we performed a comparison with published data on cytokine levels in congenital HCMV infection and in mouse models of congenital CMV infection (Table 2). Our results are aligned with the established knowledge base, confirming alterations in all cytokines and chemokines previously identified at the protein level upon MCMV infection: TNF-α, IFN-γ, IL-1β, IL-6, IL-18, CCL2, and CCL5.

In conclusion, we detected significant changes in cytokine and chemokine expression patterns in a mouse model of congenital MCMV infection, and the congruency of results with the existing knowledge base enabled us to leverage the data for a deeper understanding of the intricate cytokine and chemokine dynamics during CMV infection.

### 3.2. Harmonizing Immune Cell Attraction and Antiviral Clearance in the Brain

Congenital CMV infection is the major infectious factor associated with persistent neurodevelopmental abnormalities in newborns. Thus, our focus on the brain originates from its susceptibility to CMV during this crucial period, leading to subsequent infiltration of immune cells. Notably, all the available literature data on the recruitment of innate and adaptive immunity through soluble mediators in models of congenital infection are directed towards the brain [14,20].

**Figure 1:**
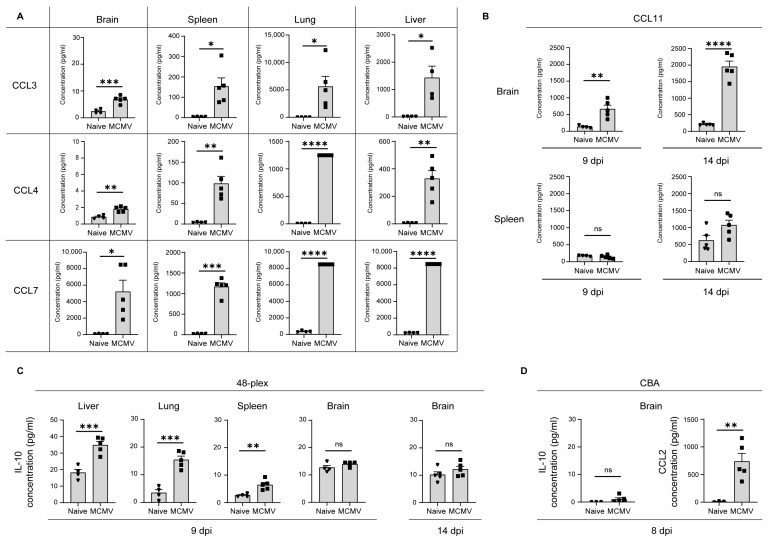
Specific brain responses in newborn CMV-infected mice. (**A**–**C**). C57BL/6J mice infected on postnatal day 1 underwent organ harvesting and lysis at specified timepoints. Cytokines were measured by ProcartaPlex. (**A**) CCL3, CCL4, and CCL7 concentrations at 9 dpi in C57BL/6J mice highlight robust immune responses. (**B**) CCL11 concentration in the brain and spleen at 9 and 14 dpi of C57BL/6J mice illustrates distinct chemokine dynamics. (**C**) IL-10 concentrations at 9 and 14 dpi in various organs and in the brain of C57BL/6J mice. (**D**) BALB/c mice were infected on postnatal day 0; at 8 dpi, IL-10 and CCL2 concentrations were confirmed by CBA kit. All concentrations are expressed as pg/mL. Mean values + SEM are shown (n = 4–5). Unpaired two-tailed Student’s test was used. A value of *p* > 0.05 was considered not statistically significant (ns): *, *p* < 0.05; **, *p* < 0.01; ***, *p* < 0.001; ****, *p* < 0.0001.

Proinflammatory proteins TNF-α, IFN-γ, IL-1β, IL-6, IL-18, CCL2, and CCL5 have been previously documented by other studies (Table 2). This manuscript discusses additional proinflammatory and immune cell attractant molecules for the first time. For instance, CCL3, CCL4, and CCL7 exhibit a marked increase consistently across all examined tissues (Figure 1A) and maintain elevated levels at 14 dpi in both analyzed organs, the brain, and spleen (Supplement Figure S1). In contrast, a distinctive elevation of CCL11 is observed in the brain at 9 dpi, and CCL11 maintains elevated levels in the brain even at 14 dpi (Figure 1B). However, no significant change in CCL11 was observed in the spleen, lungs, or liver (Table 1, Figure 1B).

Focusing on anti-inflammatory cytokines crucial for regulation of the immune response, IL-10 revealed another distinctive pattern. While IL-10 exhibited elevated levels in all tissues at 9 dpi, an exception was observed in the brain (Figure 1C). This pattern persisted at 14 dpi, indicating that the lack of IL-10 increase in the brain at 9 dpi could not be attributed to a later kinetic peak observed in the brain tissue [15]. Previous studies in adult IL-10 ko mice emphasized the critical role of IL-10 in controlling MCMV brain infection, contrasting with the lack of brain inflammation in wild-type animals. Studies by Mutnal [39] and Mandaric [40] highlighted the consequences of IL-10 absence, emphasizing pathological neutrophil infiltration into MCMV-infected brains and faster control of lytic viral replication at the expense of immunopathology. Although IL-10 protein levels were not previously determined in the congenital model of infection, transcript levels at earlier time points suggested its potential increase [37]. Therefore, the observed absence of changes in IL-10 protein level in the brain during congenital infection in our study warranted independent validation. Our confirmation using a CBA Mouse Inflammation Kit (BD) and another mouse strain (BALB/c) confirmed absence of IL-10 induction in the brain upon CMV infection at 8 dpi (Figure 1D). Concurrently, CCL2 exhibited the expected increase. Among the 47 molecules whose expression was determined, only CXCL5, a participant in neutrophil-mediated attraction, exhibited a significant decrease in the brain at 14 dpi upon CMV infection (Table 1 and Figshare 1 (https://figshare.com/s/83ba2568c37ebf2eabe8)). While the mechanistic understanding of the cytokine dynamics in the brain exceeds the scope of this manuscript, one can speculate that the decrease in CXCL5 may reduce the influx of neutrophils, potentially avoiding the need for an IL-10 increase that could have a negative impact on viral clearance from the brain.

**Figure 2:**
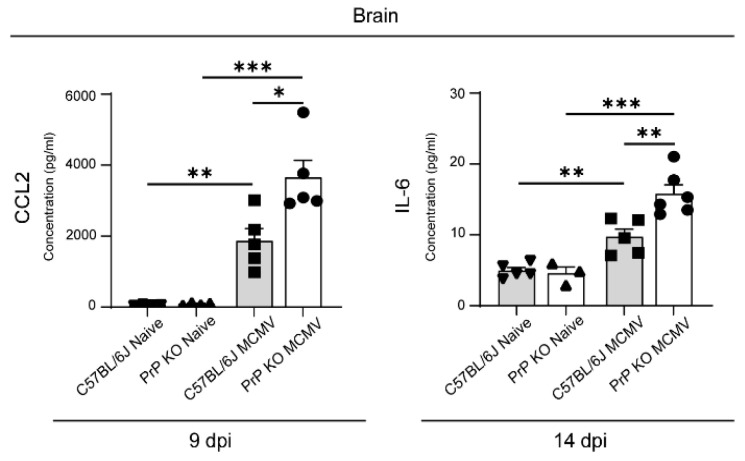
IL-6 and CCL2 levels in the brains of MCMV-infected mice. PrP ko and C57BL/6J mice were infected on postnatal day 1. Brain tissue was harvested and lysed at indicated time points. CCL2 and IL-6 concentrations were measured at 9 and 14 dpi, revealing a significant increase in PrP ko mice. All concentrations are expressed as pg/mL. Mean values + SEM are shown (n = 3–6). Unpaired two-tailed Student’s test was used. *, *p* < 0.05; **, *p* < 0.01; ***, *p* < 0.001.

We then refocused on the paradigmatic proinflammatory molecules in the brain, which have previously been shown to be upregulated and are expected to be tightly regulated. In this study, we investigated IL-6 and CCL2 levels in the brains of prion protein cellular knockout (PrP ko) mice. Previous research on PrP ko mice undergoing inflammatory processes, such as brain injury, experimental autoimmune encephalomyelitis (EAE), and brain infection with the encephalomyocarditis virus variant B (EMCV-B), has demonstrated that the absence of PrP leads to heightened neuroinflammatory damage [41,42,43]. Additionally, LPS-challenged PrP ko mice revealed a skewed outcome to a higher proinflammatory cytokine response such as IL-6 [44]. The model chemokine CCL2 is recognized for its pivotal role in CMV infection within the brain and its association with PrP in viral infections such as HIV in the brain [45], while IL-6 exhibited its influence on viral influenza infection in PrP ko mice [46]. We show here that both IL-6 and CCL2 levels were significantly increased in the brains of PrP ko mice infected with MCMV (Figure 2). These findings underscore the importance of well-controlled responses in the brain to keep inflammation under control, achieved by orchestrated effects of multiple players, including PrP, which all contribute to this intricate regulatory function.

Several immune regulatory cytokines, not significantly different between the naive and infected groups at 9 dpi in the brain, were statistically elevated in the MCMV group at 14 dpi, as highlighted by our study. Specifically, 16 interleukins (Table 1), chemokine CXCL10, and additional analytes like BAFF and BTC were significantly increased at 14 dpi compared with 9 dpi (Supplementary Figure S2). As illustrated, chemokine CXCL10, known to play an essential role in recruiting NK cells to the brain, and IL-12, a critical regulator of NK cell activation [14,47,48], were now shown to be upregulated at the protein level in CMV brain homogenates. To strengthen our findings, which were largely dependent on one multiplex analysis, we conducted a repeat of an independent experiment using 14 dpi brains from another set of mice. In Supplementary Figure S3, we present confirmation of the obtained analyte level significances for all analytes shown in the brain at 14 dpi, in the order presented in the manuscript: CCL3, CCL4, CCL7, CCL11, IL-10, CCL2, IL-6, BAFF, BTC, and CXCL10.

To conclude, the observed increases in numerous cytokines, particularly those with proinflammatory and chemoattractant properties, contribute to the establishment of neuroinflammation during CMV infection. The detection of these changes at both 9 and 14 dpi provides valuable insights into the evolving immune responses against CMV in the immature brain. Additionally, subtle differences in cytokine profiles between the brain and other organs are depicted, adding a new layer to our understanding of inflammatory response following MCMV.

### 3.3. MCMV Infection in the Periphery Induces Strong Cytokine and Chemokine Response

The majority of studies on congenital CMV infection typically focus on cytokine screening in the whole brain or specific brain regions (e.g., cerebellum) [13,14,17,36]. In this study, we extended these measurements by analyzing other organs affected by CMV’s broad tropism, all of which are also clinically relevant.

**Figure 3:**
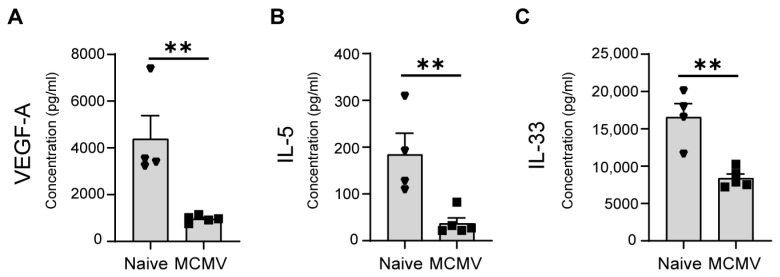
Cytokine dynamics in lungs of MCMV-infected mice. Mice were infected on postnatal day 1 (C57BL/6J). Lung tissue was harvested and lysed at 9 dpi. Concentrations of cytokines were measured by ProcartaPlex, revealing significant decreases in (**A**) VEGF-A, (**B**) IL-5, and (**C**) IL-33 levels. All concentrations are expressed as pg/mL. Mean values + SEM are shown (n = 4–5). Unpaired two-tailed Student’s test was used. **, *p* < 0.01.

Complications related to lung tissue mainly manifest in immunocompromised patients, although HCMV pneumonitis, while less frequent, poses a serious threat to congenitally infected neonates [49]. An unfortunate consequence of HCMV pneumonitis is respiratory deterioration, often attributed to immunopathology [50,51]. Our study measured a reduction in VEGF-A (Figure 3A), not previously known for MCMV, but aligning with findings in fetal and neonatal rat lung tissues infected with E. coli, where decreased VEGF was associated with morphological lung abnormalities and severe consequences similar to those observed in preterm infants with bronchopulmonary dysplasia and pneumonitis [52]. Furthermore, our analysis of lungs at 9 dpi revealed a significant increase in most cytokines and chemokines, with only IL-5 and IL-33 showing a notable decrease in infected mice (Table 1). The substantial drop in IL-5 (Figure 3B) raises considerations, as studies have implicated increased IL-5 in the recovery phase following influenza infection in the lung [53]. IL-33, known to regulate IL-5 production [53], also shows a significant decrease in MCMV-infected lungs (Figure 3C), suggesting further negative implications for immune response modulation in the context of lung MCMV infection recovery.

**Figure 4:**
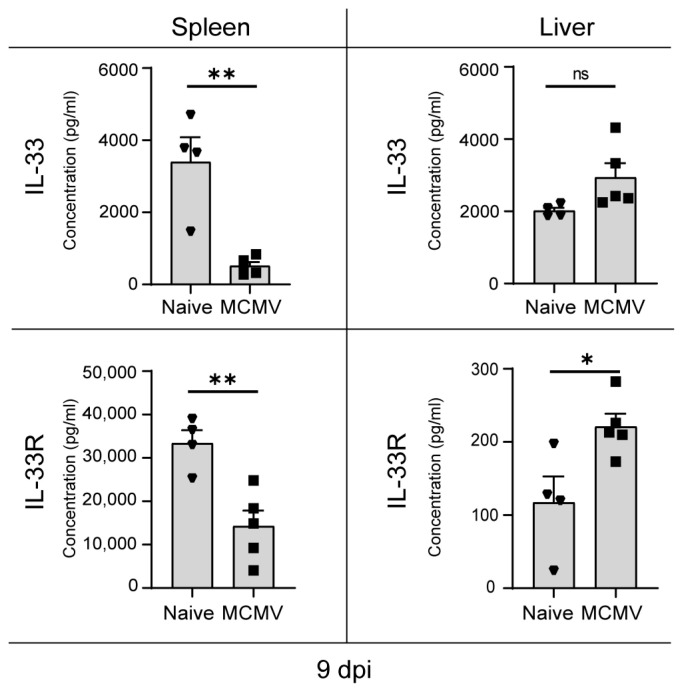
Differential IL33/IL33R dynamics in MCMV-infected mice. C57BL/6J mice were infected on postnatal day 1. Liver and spleen tissue was harvested and lysed at 9 dpi and concentrations of IL33 and IL33R analytes were measured by ProcartaPlex. All concentrations are expressed as pg/mL. Mean values + SEM are shown (n = 4–5). Unpaired two-tailed Student’s test was used. A value of *p* > 0.05 was considered not statistically significant (ns): *, *p* < 0.05; **, *p* < 0.01.

IL-33, together with its receptor IL33R, measured here in its soluble form, merits attention as a rare analyte significantly upregulated in the liver and downregulated in other tissues (lungs and spleen) (Figure 3c and Figure 4). Hepatitis in congenital CMV, although not rare, does not pose a life-threatening risk. Recent studies indicate jaundice as the most common clinical feature, accompanied by anemia, leukocytosis, and monocytosis, with patients recovering without the administration of ganciclovir The significant rise in IL-33/IL-33R concentrations, prominently observed in the liver (Figure 4), is intricately linked to the discovery that the IL-33/IL-33R signaling axis effectively mitigates MCMV-induced liver pathology through the facilitation of Treg recruitment [54]. This immunoregulatory role of IL-33/IL-33R, uniquely concentrated in the liver of MCMV-infected mice, has emerged as a robust mechanism pivotal for suppressing MCMV-induced immunopathology. The liver-specific response, which acts as a defense against liver-related complications, now indicates its presence in neonates as well.

**Figure 5:**
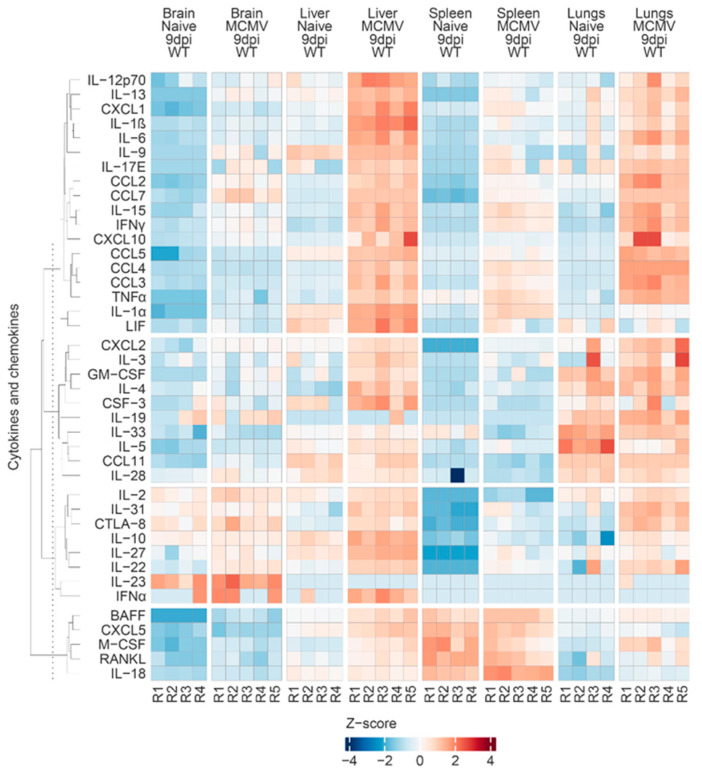
Clustered heatmap of cytokine and chemokine concentrations at 9 dpi across different organs in naïve and MCMV-infected mice. Analytes with similar expression profiles across all samples were divided into four groups, and the log(x + 1)-transformed and scaled concentration data for each molecule were visually summarized using the R computing environment, as described in Materials and Methods.

To gain a deeper insight into the organ-specific cytokine expression landscapes, we utilized K-means clustering to identify groups of cytokines that share comparable expression profiles in response to MCMV infection at nine days postinfection (Figure 5). In our selection featuring four clusters, the heatmap visually represents distinct clusters in line with the chosen K value. The first cluster depicts molecules consistently upregulated in all tissues at 9 days. As expected, cytokines such as CCL3, CCL4, and CCL7 were categorized in the first cluster, while CCL11 was placed in the second cluster due to its specific increase in the brain. Considering CCL11’s participation in neuroinflammation and its impact on postCOVID-19 neurological complications [55], such information could be of interest. The assessment of CCL11 in congenital CMV infection is limited. This chemokine has not been extensively studied in this context, and, in the specific case of amniotic fluid from congenitally infected fetuses, it was measured as 0 using the in-house multiplexed bead-based assay [38]. The chemokine CCL3 is well-documented in inflammation induced by viral infections [56]. In the context of MCMV, several findings highlight the role of macrophages contributing to CCL3 production, particularly in the liver (as reviewed by [57]). In the congenital MCMV model, we observed significant upregulation of CCL3 not only in the liver but also in other organs. This aligns with findings on CCL3 and CCL7, both CCR2-binding chemokines, reported to be upregulated upon MCMV infection in adult animals, in the bone marrow and blood, suggesting a general response [58]. Regarding CCL4, limited data in CMV indicate its potential secretion by CD4+ T cell populations specific for HCMV [59]. We measured elevated CCL4 levels in both brain and spleen tissues 14 dpi compared with 9 dpi, but further investigation is needed to determine the potential contribution of CD4 T cells to these levels.

Although the provided heatmap can offer insights into the cytokine patterns, it is important to note a drawback of this heatmap presentation. It takes into account inherent differences in tissue cytokine levels among uninfected organs, including the brain, lungs, spleen, and liver, impacting the visibility of organ-specific changes. Therefore, we provide a Figshare 2 (https://figshare.com/s/d0afb41451bc12050892) for the table, including all levels of measured molecules, allowing for customized heatmap exploration of the possible variation in the number of clusters or comparisons through 9 and 14 dpi in the brain or spleen for the interests of others. Additionally, we offer a detailed Methods and Materials section explaining how the chosen heatmap (9 dpi, all organs, all cytokines and chemokines, four clusters) was generated. In our heatmap representation, we observe a generally well-correlated pattern among different organs, with an anticipated increase in the periphery at the 9 dpi time point.

## 4. Conclusions

Inflammatory responses during congenital CMV infection involve the activation of immune cells and the expression of various proinflammatory cytokines [10,13,14,15,16,17,18,19]. Traditionally, pivotal players like IFNγ and TNFα have been the focus of antiviral defense [14,20,60,61,62]. However, our study employed a mouse model to comprehensively analyze 32 cytokines, 10 chemokines, 3 growth factors/regulators, and 3 soluble receptors simultaneously, using a 48-plex xMAP panel. We describe the first usage of Luminex to define cytokine/chemokine expression in various tissues in a congenital MCMV infection model. While cytokines have been measured in sera and brain tissue of MCMV-infected newborn mice, most researchers used ELISA-based protein concentration assays for selected targets or detection based on RNA levels (Table 2). Regarding human samples, the analysis of blood secretome after CMV-specific stimulation was previously investigated using a multiplex detection technique [63]. However, these samples were from adult transplantation patients, whereas our research strictly aims to profile the expression of soluble immune mediators in congenitally infected tissues. In this regard, there is a study on cytokine/chemokine immune response that includes research based on an in-house multiplexed bead-based assay of amniotic fluid from HCMV-infected fetuses [38]. Several publications have used Luminex to detect cytokines in mouse tissue in the context of viral infection, including lung tissue in SARS-CoV-2 [64], influenza A virus [65], a mouse model of human respiratory syncytial virus infection [66], and also liver tissue in LCMV infection-induced viral hepatitis [67]. One limitation of our study is the use of a BAC-derived MCMV strain carrying a mutation in the MCK-2 viral-encoded chemokine, which is known to influence viral entry and immune infiltrates, as reviewed in [68]. In the neonatal model, it has been demonstrated thus far that MCK-2 influences viral tropism in the lungs [69], though cytokines were not measured in comparison, indicating a potential avenue for future investigation. In our examination of the liver, lungs, spleen, and brain at 9 dpi, with additional scrutiny of the latter two organs at both 9 and 14 dpi, we successfully detected 47 out of 48 analyzed molecules. Our findings demonstrated statistical elevation in 16 analytes across all organs at 9 dpi. Notably, at 14 dpi, nine cytokines and six chemokines sustained statistical elevation in both the spleen and brain. In the brain, our analysis identified 25 induced molecules at 9 dpi, with an additional 8 exhibiting statistically elevated responses at 14 dpi. Venturing beyond traditional markers, we explored proinflammatory and immune cell attractant molecules, such as CCL3, CCL4, CCL7, and CCL11, each displaying distinct elevation patterns. Notably, 16 interleukins, excluding IL-10, exhibited elevation in the brain during infection, contrary to expectations based on previous studies. The heightened levels of IL-6 and CCL2 in PrP ko underscored intricate regulatory dynamics during CMV infection in the brain. Analyzing IL-33/soluble IL-33R levels offered insights into the IL-33/IL-33R signaling axis’s potential role in mitigating MCMV-induced liver pathology in the congenital model. Our results provide a detailed immune soluble mediators database, showcasing significant changes in cytokine and chemokine expression patterns in a mouse model of congenital MCMV infection. Overall, these findings contribute to a deeper understanding of the complex cytokine and chemokine dynamics in various organs, shedding light on potential avenues for further research and therapeutic strategies.

## Figures and Tables

**Table 1:** Cytokine and chemokine expression in congenitally infected C57BL/6J mice at 9 and 14 dpi. C57BL/6J mice were infected with MCMV. At 9 or 14 dpi, mice were sacrificed, and brain and spleen were harvested at both time points, while the liver and lungs were harvested at 9 dpi. Analyte concentrations in the infected group were compared to the uninfected control group, and if significantly altered upon infection, they are shaded: green if elevated and red if downregulated. At 9 dpi, the groups of mice consisted of 4 C57BL/6J naive and 5 C57BL/6J MCMV-infected mice, and for 14 dpi, 5 C57BL/6J naive and 5 C57BL/6J MCMV-infected mice were included. The Figshare 2 (https://figshare.com/s/d0afb41451bc12050892) provides raw data (pg/mL) on the obtained concentrations in each organ of individual mice.

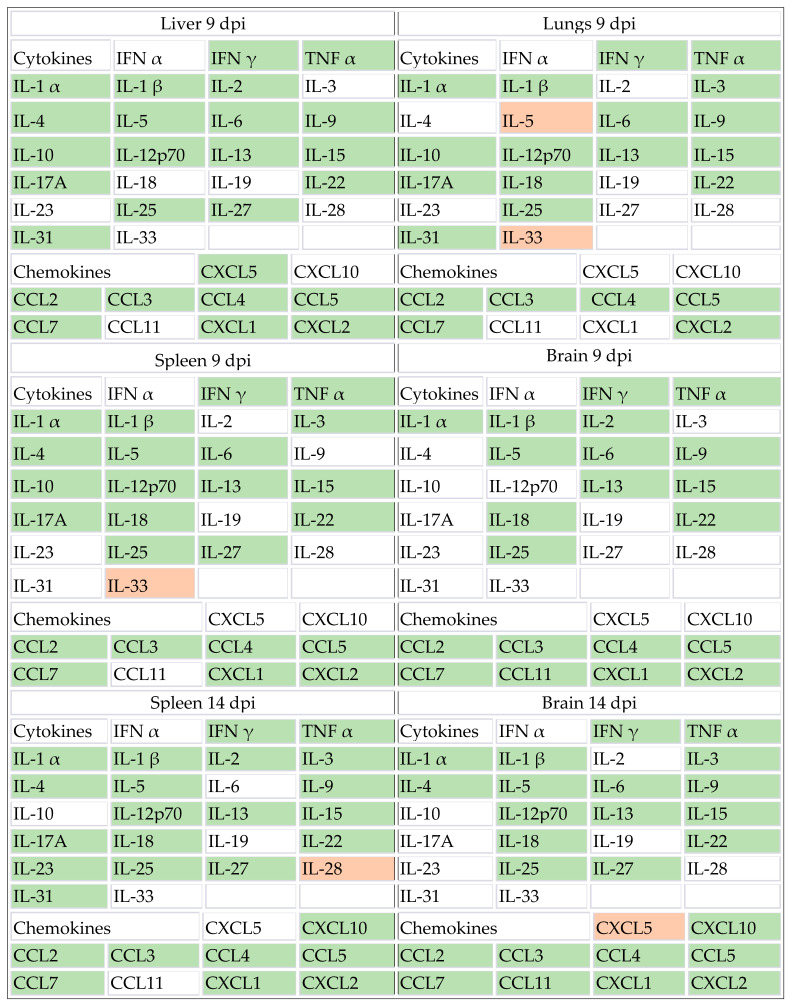

**Table 2:** Overview of available data regarding MCMV and HCMV infection. This table supplies cross-validation data for 14 identified molecules, the only available pre-existing information within our set of 47 analytes. Data are categorized based on the type of prior analysis (yellow for transcript and green for proteins). The symbol “+” signifies cases in which the molecule was identified in the literature, consistently indicating an elevated value during infection in all instances.

MCMV								HCMV	
Newborn							Fetal	Amniotic Fluid	
	This Manuscript	Koontz [13]	Kosmac [17]	Seleme [20]	Kvestak [14]	Zhou [36]	Sakao-Suzuki [37]	Bourgon [38]	Scott [19]
IFNα	+	+					+		
IFNγ	+		+	+	+	+	+		
TNFα	+	+	+	+	+	+	+		
IL-1β	+	+		+		+	+		+
IL-6	+				+		+		
IL-10	+						+		
IL-12	+								+
IL-17	+								+
IL-18	+					+		+	
CCL2	+			+			+		+
CCL4	+								+
CCL5	+	+		+					
CXCL10	+	+			+			+	+
CXCL9					+				

## Data Availability

The Figshare 1 (https://figshare.com/s/83ba2568c37ebf2eabe8) offers a detailed molecule-by-molecule breakdown, facilitating a thorough evaluation of all 48 identified molecules. The Figshare 2 (https://figshare.com/s/d0afb41451bc12050892) provides raw data (pg/mL) on the obtained concentrations in each organ of individual mice at both time points.

## Supplementary Materials

The following supporting information can be downloaded at: https://www.mdpi.com/article/10.3390/v16020208/s1. The manuscript is accompanied by three supplementary figures within the supplemental material: Figure S1. Prolonged immune activation: CCL3, CCL4, and CCL7 levels remain elevated at 14 days postinfection in C57BL/6J mice; Figure S2. BAFF, BTC, and CXCL10 levels in the brains of MCMV-infected mice; Figure S3. Biological replicate of cytokines and chemokines at 14 dpi in C57BL/6J mice.
