# Supplementary material for: Comprehensive Analysis of Soluble Mediator Profiles in Congenital CMV Infection Using an MCMV Model
Source: Viruses. 2024 Jan 30;16(2):208. doi: 10.3390/v16020208 (PMC10891658; doi:10.3390/v16020208)
Supplement: Supplementary file 1 [file viruses-16-00208-s001.zip › viruses-2813683-supplementary.pdf]

viruses

Supplementary Materials:

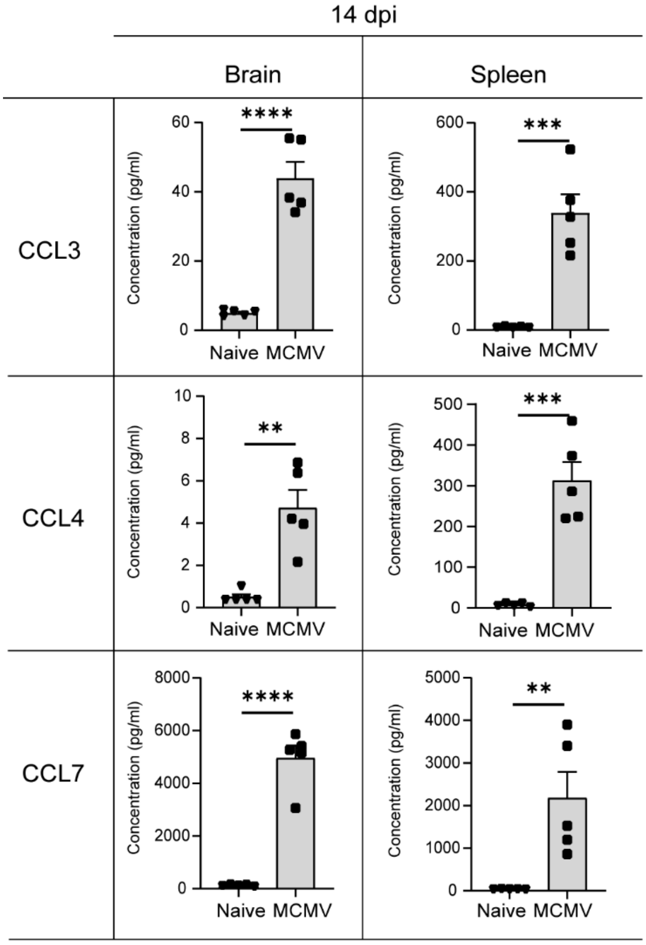

Supplement Figure S1. Prolonged immune activation: CCL3, CCL4, and CCL7 levels remain elevated at 14 days post-infection in C57BL/6J mice. C57BL/6J mice infected on postnatal day 1 underwent organ harvesting and lysis at 14 dpi. CCL3, CCL4, and CCL7 concentrations are expressed as pg/ml. Mean values + SEM are shown (n = 4–5). Unpaired two-tailed Student's test was used. A value of $P > 0.05$ was considered not statistically significant (ns); **, $P < 0.01$; ***, $P < 0.001$; ****, $P < 0.0001$.

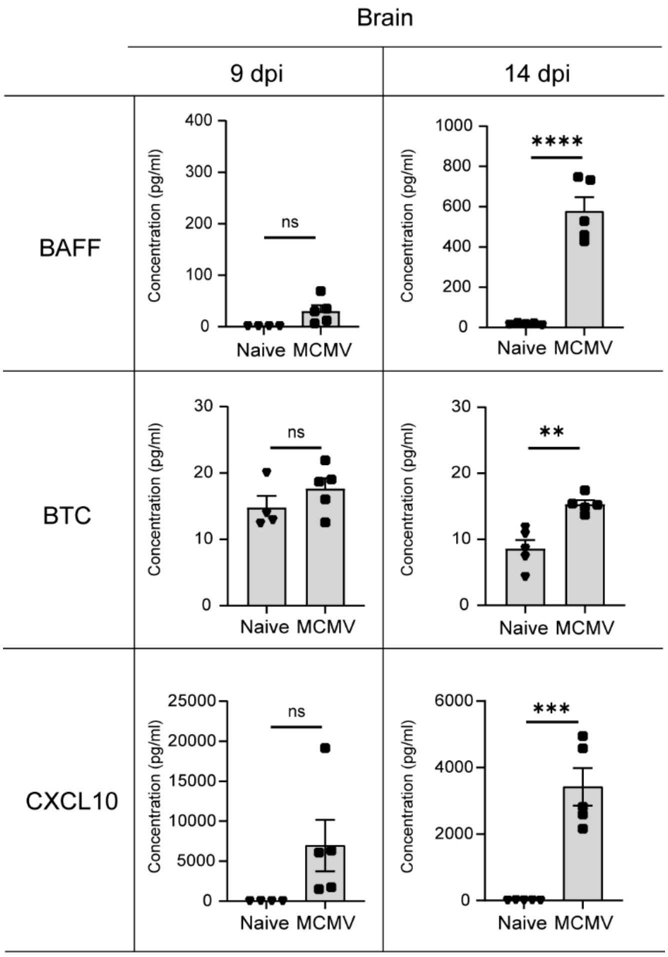

Supplement Figure S2. BAFF, BTC and CXCL10 levels in the brains of MCMV-infected mice. C57BL/6J mice were infected on postnatal day 1. Brain tissue was harvested and lysed at indicated timepoints. BAFF, BTC and CXCL10 concentrations were measured at 9 and 14 dpi. All concentrations are expressed as pg/ml. Mean values + SEM are shown (n = 4–6). Unpaired two-tailed Student's test was used. A value of $P > 0.05$ was considered not statistically significant (ns); **, $P < 0.01$; ***, $P < 0.001$.

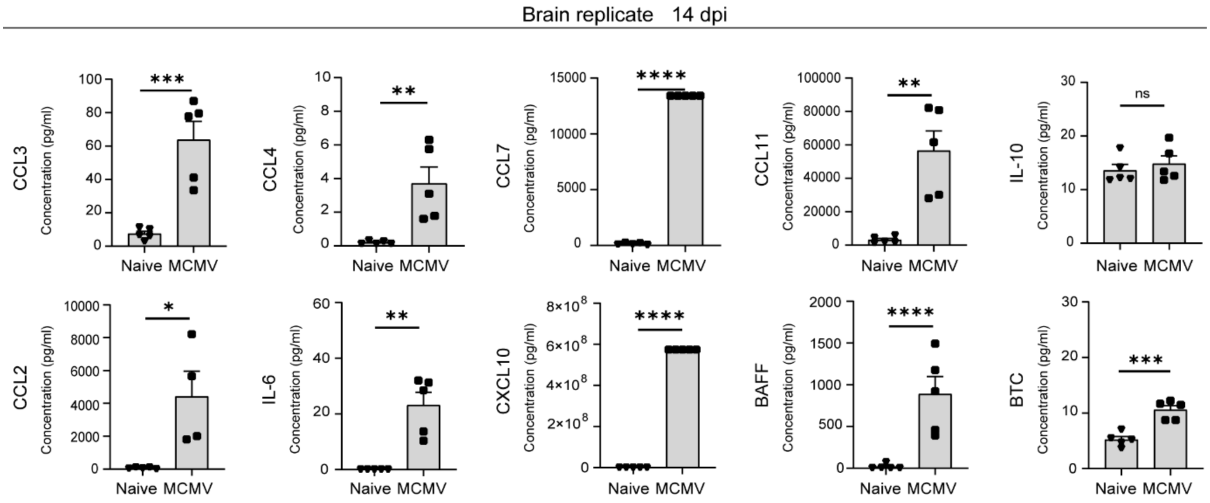

Supplement Figure S3. Biological replicate of cytokines and chemokines at 14 dpi in C57BL/6J mice. C57BL/6J mice infected on postnatal day 1 underwent organ harvesting and lysis at 14 dpi. All concentrations are expressed as pg/ml. Mean values + SEM are shown (n = 4–5). Unpaired two-tailed Student's test was used. A value of P > 0.05 was considered not statistically significant (ns); *, P < 0.05; **, P < 0.01; ***, P < 0.001; ****, P < 0.0001.
